# New targets for new therapeutic approaches

**DOI:** 10.1186/s13054-014-0669-8

**Published:** 2014-12-13

**Authors:** Bruno François

**Affiliations:** Service de Réanimation Polyvalente, CHU de Limoges, 2 Avenue Martin Luther King, 87042 Limoges, Cedex France; INSERM, CIC 1435, CHU Dupuytren, 2 avenue Martin Luther King, 87042 Limoges, Cedex France

## Abstract

Because of its resistance profiles, *Pseudomonas aeruginosa* remains probably one of the challenging bacteria responsible for ventilator-associated pneumonia in the ICU. Nevertheless, a much better understanding of its mechanism of virulence, such as the type 3 secretion system that can also impact on resistance, gives some opportunities for management improvement. The most promising approach is probably the production of monoclonal antibodies that enable not only more targeted treatments but also development of some early preemptive approaches at the time of colonization through real-time diagnosis.

Ventilator-associated pneumonia (VAP) is probably one of the last infections remaining challenging in the ICU, unlike sepsis and other ICU-specific infections for which management is much better defined, and Sawa and colleagues bring some new understanding and therapeutic options [1]. In this specific clinical setting, two microorganisms are predominantly involved – *Staphylococcus aureus* and *Pseudomonas aeruginosa* – both sharing the same resistance issues and treatment controversies.

Owing to its larger genome, encoding extreme phenotypic versatility, motility, virulence, and persistence factors as well as its intrinsic and acquired drug resistance, *P. aeruginosa* is possibly the most interesting of the opportunistic bacteria. In this issue of *Critical Care*, Sawa and colleagues demonstrate that *P. aeruginosa* has the capability of producing numerous toxins, of which ExoU seems to play a key role. These authors convincingly illustrate the potential link between *P. aeruginosa* virulence and resistance, with a special emphasis on ExoU-associated virulence. As a major component of the type 3 secretion system (T3SS), which is responsible for the injection of ExoU and various cytotoxic virulence factors into host cells, PcrV appears to be a promising target for future VAP prevention agents. Importantly, clinical studies have previously suggested a relationship between *P. aeruginosa* T3SS gene expression in clinical isolates (blood cultures) and outcome [2]. Additional virulence factors are also frequently involved in *P. aeruginosa* VAP. Quorum sensing-regulated virulence factor rhamnolipids have been identified in patients with *P. aeruginosa* VAP and its inhibition appears to be an alternative therapeutic strategy [3]. Psl is a serotype-independent exopolysaccharide implicated in initial colonization and so-called immune evasion [4]. Of note, these various mechanisms of virulence/resistance may interact, such as Psl and T3SS expression, and jointly play a leading role in the development and persistence of *P. aeruginosa* infections, including VAP [5].

While the *P. aeruginosa* mechanism of action is increasingly decrypted, diagnostic methods have tremendously improved over the last decade, moving from time-consuming conventional culture towards real-time bedside diagnosis. Reverse transcription polymerase chain reaction is bringing online diagnosis to the clinical grounds and promises to enable early targeted therapy in the near future. In addition, continuous technical proceedings facilitate analysis processes and open new perspectives on the routine use of these novel diagnostic approaches for a bedside analysis directly on any lung biological sample (bronchoalveolar lavage, endotracheal aspirates, and so forth). Undoubtedly, *P. aeruginosa* virulence determinants being better understood, these newly developed diagnostic techniques promise to simultaneously provide the front-line intensivist with valuable information on bacterial virulence and even the drug-resistance profile.

In parallel, VAP management has also improved dramatically. The major issue of induced resistance by antibiotics definitely supports the recommended bundles to prevent VAP, but other benefits are also the prioritization of targeted antibiotic therapy and reduced treatment duration [6]. The most promising therapeutic perspective is the identification of specific *P. aeruginosa* targets for specific monoclonal antibody (mAb) development.

Several anti-pseudomonas mAbs are currently developed for clinical use and recent publications have demonstrated their potential clinical value for VAP treatment [7,8]. The combined early *P. aeruginosa* infection diagnosis capability using real-time techniques such as polymerase chain reaction and mAb availability have paved a new road for the preemptive therapeutic approach in *P. aeruginosa* VAP and introduced a paradigm shift in critical care medicine. With colonization preceding infection (also sometimes referred to as disease) in nearly all cases, the challenge is now to confirm the validity of such an approach. We reported recently convincing results in a phase II trial that tested a preemptive mAb therapeutic approach targeting PcRv in ICU *P. aeruginosa* colonized patients [8]. In addition, given their complementary roles, a combination of the anti-Psl and anti-PcrV mAbs as a single drug candidate (MEDI3902; MedImmune, AstraZeneca, Gaithersburg, MD, USA) could increase the benefit in a preemptive VAP approach against *P. aeruginosa*. To test this hypothesis, trials should start soon within the ND4BB program (supported by the Innovative Medicines Initiative) that made the fight against multiresistant bacteria, and especially *P. aeruginosa*, one of its main objectives. A similar preemptive approach targeting *S. aureus* alpha toxin with a long half-life mAb (MEDI4893; MedImmune, AstraZeneca) is already underway in this program.

In conclusion, it is probably not unrealistic to think that in the very near future we should be able to rapidly detect the presence of *P. aeruginosa* in the lung or lower respiratory tract and at the same time assess both drug-resistance and virulence potential for a preemptive strategy using highly specific mAbs to neutralize key targets in bacterial virulence, persistence and resistance to therapy. This theranostic approach focused on new targets could undoubtedly be the magic bullet to reduce VAP incidence, as well as the VAP-associated morbidity and mortality.

## Abbreviations

mAb: monoclonal antibody
T3SS: type 3 secretion system
VAP: ventilator-associated pneumonia
